# A Web-Based Intervention to Encourage Walking (StepWise): Pilot Randomized Controlled Trial

**DOI:** 10.2196/resprot.4288

**Published:** 2016-01-25

**Authors:** Elaine Anne Hargreaves, Nanette Mutrie, Jade Dallas Fleming

**Affiliations:** ^1^ School of Physical Education, Sport & Exercise Sciences University of Otago Dunedin New Zealand; ^2^ Physical Activity for Health Research Centre Institute for Sport, Physical Education and Health Sciences University of Edinburgh Edinburgh United Kingdom

**Keywords:** physical activity, behavior change strategies, pedometer, self-monitoring, step goal walking program

## Abstract

**Background:**

Despite Internet-based interventions that incorporate pedometers with appropriate goal-setting processes and other theoretically-based behavior change strategies being proposed as a means of increasing walking behavior, few have incorporated all of these key features or assessed maintenance of behavior change.

**Objective:**

The objective of our study was to investigate the effect of a 12-week pedometer step goal walking program individually tailored to baseline step counts, combined with an interactive support website for step counts, health parameters and motivation over 12 and 24 weeks.

**Methods:**

Low active participants (mean [SD] 46.2 [11.2] years) were randomly assigned to the Stepwise (SW) intervention group (n=49) or a comparison (CP) group (n=48). SW received a pedometer, step goal walking program and access to the SW website (containing interactive self-monitoring and goal feedback tools, motivational messages and action and coping planning strategies). CP received a pedometer and locally available physical activity information. Step counts, BMI, resting heart rate, blood pressure and glucose, cholesterol and triglyceride levels, psychological well-being, perceived health, self-efficacy and self-determined motivation were measured at baseline, 12 and 24 weeks.

**Results:**

Linear mixed model analysis found that both groups’ step counts increased from baseline to week 12 (β = 11,002, CI 5739-16,266, *P*<.001) and 24 (β = 6810, CI 1190-12,431; *P*=.02). Group step counts were significantly different at week 24 with SW taking 8939 (CI 274-17604, *P*=.04) more steps compared to CP. Compared to baseline, both groups had improved triglyceride levels (0.14 mmol/L, CI -0.25 to -0.02, *P*=.02) at week 12, decreased diastolic blood pressure (4.22 mmHg, CI -6.73 to -1.72) at weeks 12 and 24 (3.17 mmHg, CI -5.55 to -0.78), improved positive (β = .21, CI 0.03-0.38, *P*=.02) and negative affect (β = -.15, CI -0.28 to -0.03, *P*=.02) at week 12, and perceived health at week 12 (β = 6.37, CI 2.10-10.65, *P*=.004) and 24 (β = 8.52, CI 3.98-13.06, *P*<.001). Total cholesterol increased at week 12 (0.26 mmol/L, CI 0.099-0.423, *P*=.006) and week 24 (0.38 mmol/L, CI 0.20-0.56, *P*<.001). Repeated measures ANOVA found motivation for walking improved from baseline with higher task self-efficacy (*P*<.001, η^2^ = .13) and autonomous motivation (*P*<.001, η^2^=.14) at weeks 12 and 24 and decreased controlled motivation (*P*=.004, η^2^=.08) at week 24.

**Conclusions:**

Both groups had similar improvements in step counts and physical and psychological health after 12 weeks but only the SW group successfully maintained the increased step-counts 24 weeks post-intervention. This suggests the step-goal based walking program combined with Internet-based behavior change tools were important for sustained behavior change.

## Introduction

Regular moderate-intensity physical activity amounting to at least 150 minutes per week is one of the best lifestyle choices an individual can make to improve health, sustain good health and prevent ill health [1-2]. Despite the widespread benefits of engaging in an active lifestyle, participation rates remain low in the (nonclinical) general population [3-4]. Walking is regarded as the modality most likely to increase activity levels [5-6] as it is safe, inexpensive, does not require any special clothes or skills, is accessible to all socio-economic groups and can be easily incorporated into a daily routine. Walking interventions are effective in improving health based parameters [7-10]. Research suggests that walking interventions that are tailored to the individual, incorporate the motivational function of a pedometer [6], are informed by behavior change theory [11] and utilize the power of a Web-based delivery [12] can successfully increase walking behavior.

Incorporating a pedometer into a walking intervention with a nonclinical population of adults is associated with significant increases in physical activity of 2000-2500 steps per day [13-15] and this increase has been associated with clinically relevant reductions in weight and blood pressure [14]. The association with other health variables which are risk factors for cardiovascular disease, such as cholesterol, triglyceride and glucose levels, remain inconsistent [10]. Reducing cardiovascular disease risk remains an important health outcome. Although there is a positive relationship between physical activity and cardiovascular health, understanding the ability of specific pedometer interventions to demonstrate changes in a variety of health variables remains important so that health care professionals are aware of the health benefits that can be accrued when they are promoting these interventions [10]. The success of pedometer-based interventions, and interventions delivered using the Internet, comes when they are informed by behavior change theory [10,16]. As suggested by Ritterband et al [17] it is unlikely that one single theory or model can explain behavior change in an Internet intervention. Consequently, this study is underpinned by a number of key behavior change techniques (BCTs) drawn from Social Cognitive Theory [18] and Self-Regulation Theory [19] known to effect change in walking behavior [16]. Self-monitoring and feedback strategies are important to increase awareness of behavior, provide a tangible record of success, instill accountability [20-21] as well as increase confidence and reduce perceived barriers to walking [22-23]. The motivational feedback provided should be tailored to participant characteristics rather than being generic [24]. Action planning and coping planning help translate intentions into behavior [25] and are important for relapse prevention [17]. Lastly, a goal-setting component is key to a successful pedometer intervention [11]. Goal-setting strategies used in pedometer studies vary considerably and range from a generic fixed goal of 10,000 steps per day [26], a fixed increment between 1000 steps per day to 3000 steps per day over baseline [27-28], to a 10-20% increase based on the previous weeks steps [26,29]. For inactive individuals to feel motivated and confident of being successful, goals need to be individualized so they are realistic, achievable and easily adjusted when necessary [11]. Consequently, we favor the approach used by Fitzsimons et al [30]. Their 12-week walking program gradually increased weekly step goals so that by Week 7 participants are aiming to achieve 3000 steps above baseline values at least 5 days per week. This equates to 30 minutes of physical activity and meets the physical activity recommendations for health [31]. Community-based participants following the walking program showed significant increases in step counts from baseline to 12 weeks [32] and to 12 months [33]. Positive affect and perceived health also improved. This walking program has not been tested using a Web-based delivery.

Pedometer-based interventions of varied length have been conducted using Internet technology [27-29,34]. The majority provide step goals and ask individuals to log steps, but few have incorporated more than 1 or 2 key BCTs suggested as important for successful step count increases. A meta-analysis has shown that the more BCTs incorporated into an intervention, the larger the effect on behavior [16]. As well as motivating behavior change, these techniques can reduce participant attrition and sustain engagement with an intervention [35-36] which is important as website visits, and therefore exposure and engagement with the intervention, have been shown to decrease over time [12]. One exception is Richardson et al’s [28] 6-week intervention where participants with type 2 diabetes received tailored motivational messages (highlighting the benefits of exercise and how to overcome barriers), educational tips, automatically calculated goals and feedback in relation to their performance towards the goals. Results showed step counts increased by ~ 1950 steps per day. Our study differs from Richardson et al in that we are targeting a nonclinical sample of adults, our motivational messages target the building of both task and barrier self-efficacy as well as autonomous motivation, we offer a different approach to goal-setting and the study is longer in duration. Furthermore, Richardson et al’s research, similar to most Web-supported studies, did not measure maintenance of step changes following the intervention. Carr et al [34] investigated maintenance of step counts and found step counts had returned to baseline after 8 months. However, this was not a pedometer intervention; they simply used pedometers to measure physical activity behavior. The importance of investigating sustained behavior change from a Web-supported pedometer intervention has been recognized [13,37].

To overcome the limitations of previous research, we created the StepWise intervention which combines an individually-tailored step goal pedometer walking program with an interactive support website. The website allows the participants to enter their step counts, graphically see their goal achievement, obtain automated and individualized motivational messages and plan their walking activities. This pilot study consisted of a 12-week intervention with a 12-week follow-up to investigate whether the StepWise intervention would increase step counts, improve health parameters and motivation for walking compared with a comparison group in a community sample of apparently healthy adults.

## Methods

### Study Design

This pilot randomized trial compared a StepWise intervention group (SW) to a comparison group (CP) over a 12-week intervention and a 24-week follow up. The intervention was fully Web-based but the study also involved face-to-face components, specifically to collect outcome data and for the intervention and comparison procedures to be explained to participants. The trial is reported in accordance with CONSORT-EHEALTH guidelines. The research was approved by the University Human Ethics Committee in accordance with all applicable regulations (July 9th, 2012, reference 12/159).

### Participants

A targeted recruitment strategy was employed [38] to attract participants from the community who were not currently meeting physical activity recommendations (ie, participated in <150 minutes of moderate intensity physical activity per week), who were aged over 25 years (to exclude a student population) and were apparently healthy. Participants were recruited offline in September of 2012. Advertisements were placed in community newspapers, a recruitment email was sent through the internal email systems of the local University, Polytechnic, City Council, primary and high schools, and posters were placed in areas where low-active individuals would see them (eg, GP practices, supermarkets, local shops, community and church halls). To be eligible, individuals had to be able to walk, have no contraindications to participate in a moderate-intensity walking program, and have regular access to the Internet (it was presumed that participants who responded to the recruitment advert would be computer/Internet-literate). Interested participants contacted the research team via email or telephone and were given detailed information about what was involved in study participation. To screen for eligibility, participants were asked to explain what physical activity they currently participated in and whether or not they were taking any medications for health conditions. Those participants who reported taking medication for blood pressure or cholesterol were admitted to the study, but their data was not used in the analysis of those variables. Those who met the study criteria and were still interested in participating attended a baseline testing session where they completed the Physical Activity Readiness Questionnaire [39] to ensure they had no contraindications to participate in physical activity and provided informed consent after the nature and possible consequences of the study were explained.

### Measures

All measurements took place in a room at the University in the morning. Participants arrived in a fasted state having done minimal physical activity that morning. The same measurements were taken at baseline, 12 weeks, and 24 weeks.

#### Primary Outcome Measure: Step Counts

Physical activity was assessed by step counts. Participants were given a Yamax PW-610 pedometer, individually calibrated consistent with manufacturers’ guidelines and asked to wear the pedometer during all waking hours for the next 7 days and to remove it only when sleeping, bathing or during water-based activities. The screen of the pedometer was covered so participants could not see their step counts. At baseline they were encouraged to continue with the same amount of physical activity as they had been doing the previous week. One week later, the researcher removed the screen cover and step counts were recorded. The pedometer was returned to the participant to use in the study.

#### Secondary Outcome Measures

##### Health Variables

Participants were measured for height and weight in order to calculate Body Mass Index (weight in kg/height in m^2^), seated resting heart rate (measured using a Polar PE3000 heart rate monitor) and blood pressure (using a manual sphygmomanometer). A trained phlebotomist then drew a 6 ml blood sample from the participant’s arm by venipuncture and the sample was analyzed for glucose, total cholesterol, HDL cholesterol and tryglycerides. After collection the venous blood was centrifuged and the heparinized plasma was analyzed for Glucose, Total Cholesterol, HDL cholesterol and Triglycerides using a Cobas C111 analyzer (Roche Diagnostics). Staff taking these measurements and doing the analysis were blinded to group allocation. Participants completed the Positive and Negative Affect Schedule [40] to assess psychological well-being (measured on a 5-point Likert-type scale, ranging from “very slightly or not at all” to “extremely”). Positive affect reflects the extent to which the individual feels enthusiastic, active and alert, a state of pleasurable engagement. Negative affect reflects the extent to which the individual experiences subjective distress and unpleasurable engagement that subsumes a number of aversive mood states. Positive and negative affect are two distinct dimensions of affective state and are not bipolar opposites [40]. Participants also completed the Visual Analogue Scale of the Euroqol EQ-5D [41] to assess self-rated health status (measured on a 100 point scale, from “worst health you can imagine” to “best health you can imagine”).

##### Motivation

Participants completed a number of motivation questionnaires. The Behavioral Regulation in Exercise Questionnaire-2 [42] is a scale from which a measure of autonomous motivation (motivated by value attached to the outcomes of being active and the enjoyment gained) and controlled motivation (motivated by need for reward or as a result of feeling pressured to be active) was created [43-44]. Items are measured on a 5-point Likert-type scale ranging from “not true for me” to “very true for me”, The Barriers for Habitual Physical Activity Scale [45] assessed the barriers to participating in physical activity (measured on a 5-point Likert-type scale ranging from “strongly disagree” to “strongly agree”). Finally, measures of self-efficacy for walking [46] and for overcoming barriers to exercise [47] were measured on a 10-point scale from “not at all confident” to “completely confident”.

##### Intervention Use

Participants were encouraged to log into the website at least once a week. Website usage statistics were downloaded at week 12 to assess engagement and adherence to the intervention. Additionally, participants completed a questionnaire asking (1) whether they had used the SW website (yes/no), (2) how often (more than once a week, once a week, once every 2 weeks, 3-4 times over the 12 weeks, 1-2 times over the 12 weeks), and (3) to write down what aspects of the website they had found most and least useful.

### Randomization

One week after baseline testing, participants attended a second face-to-face session where they were randomized into either SW or CP groups. To ensure equal representation in the groups, randomization was stratified by gender (male or female) and age (<45years or >45years) creating 4 distinct stratification groups. Group assignment was placed inside sealed envelopes and the envelopes shuffled to produce an unpredictable sequence of group assignment. When each participant arrived, the next envelope in the pile representing that individual (male or female and <45 or >45years) was opened to reveal their group assignment. As far as we are aware, participants could not tell whether they were in the SW or CP group because all study information stated participants would be given access to a pedometer walking program and a supportive physical activity website.

### StepWise Intervention Group

The SW intervention consisted of 2 components: (1) an individualized pedometer-based walking program with weekly step goals, and (2) a website (created by the University Web development team) where individuals entered their step counts, received goal feedback, their next weekly goal (from the walking program) and tailored motivational feedback, and created a physical activity plan. At the second face-to-face session, the intervention components were discussed with each participant and the participant left with an information sheet summarizing the discussion. The same researcher met with each participant and the information discussed was standardized across all participants to ensure accuracy and consistency in the delivery of the intervention.

#### Individualized Pedometer-Based Walking Program

The walking program was structured around each participant’s baseline step counts and designed so that physical activity increased gradually [30,32-33]. By the seventh week, participants would be walking an extra 3000 steps per day over their baseline and meeting the physical activity guidelines (see Textbox 1).

**Pedometer-based incremental walking program goals.**

- Week 1: Walk an extra 1500 steps (from baseline value) on at least 3 days of the week
- Week 2: Walk an extra 1500 steps (from baseline value) on at least 3 days of the week
- Week 3: Walk an extra 1500 steps (from baseline value) on at least 5 days of the week
- Week 4: Walk an extra 1500 steps (from baseline value) on at least 5 days of the week
- Week 5: Walk an extra 3000 steps (from baseline value) on at least 3 days of the week
- Week 6: Walk an extra 3000 steps (from baseline value) on at least 3 days of the week
- Week 7: Walk an extra 3000 steps (from baseline value) on at least 5 days of the week
- Week 8: Walk an extra 3000 steps (from baseline value) on at least 5 days of the week
- Weeks 9-12: Maintain walking levels using the week 7 goal

#### StepWise Website

**Figure 1:**
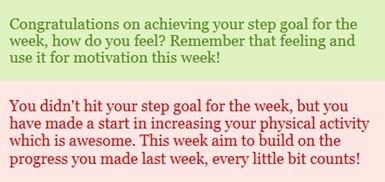
Examples of success and failure messages shown once step counts were entered.

**Figure 2:**
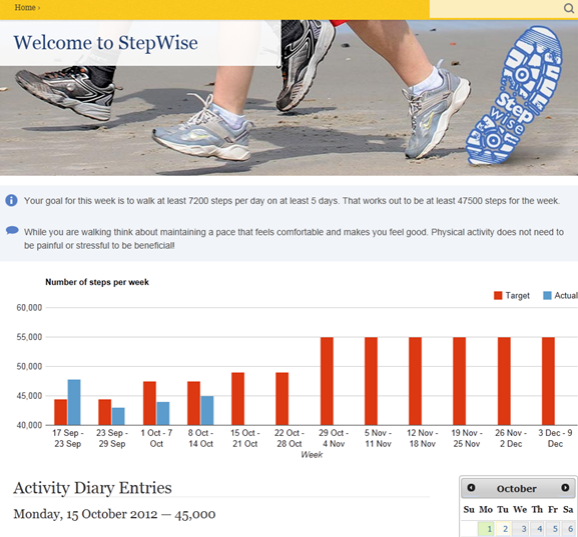
The graph showing step goals and number of steps achieved each week and the step goal for the following week along with a motivational message.

Each participant’s baseline step counts and reported barriers to physical activity (from Barriers for Habitual Physical Activity Scale) [45] were entered manually by the researcher and an automated algorithm generated the individual’s step count goal for each week (based on the walking program). Each participant created their own website log-in and password. Participants were sent an email at the end of each week prompting them to log-in to the website and enter their weekly step counts (read from the pedometer memory). The website algorithm calculated whether or not they had achieved their weekly step goal and generated a motivational message relating to whether they had been successful or unsuccessful in achieving their goal (see Figure 1 for message examples). A graph showed the step goal number and the actual steps achieved. Above the graph, the step goal number for the next week and a motivational tip to help them achieve it was displayed (see Figure 2). These motivational messages and tips (which changed each week) were created based on behavior change theory around building task and barrier self-efficacy and autonomous motivation. Finally, participants were prompted to use the activity diary feature to write an action plan for how they would achieve their step goals and a coping plan to overcome any barriers they might face in trying to achieve that plan.

#### Twelve-Week Follow-Up

At the end of the 12-week intervention, participants met with the researcher and were encouraged to maintain their new level of physical activity over the following 12 weeks. They kept the pedometer and were told they could continue to access and use the StepWise website to receive step goals. To support maintenance of behavior change, there was a discussion of strategies relating to relapse prevention. This involved encouraging the participant to identify factors that could interfere with their being able to continue their new walking behavior and thinking of strategies they could put in place to overcome these factors so they could remain active. In the 12-week follow-up period, participants were sent 2 emails reinforcing the messages that had been discussed.

#### Comparison Group

Comparison group (CP) participants were given a pedometer and informed of the public health guidelines for physical activity (150 minutes of weekly moderate intensity physical activity) and how the guidelines translated into pedometer steps. Participants were shown the MoveMe website [48] a noninteractive city-specific physical activity website which provides information on physical activity and local physical activity opportunities. In comparison to the StepWise website, the MoveMe website does not contain any interactive features known to encourage physical activity behavior change. Participants were encouraged to access the MoveMe website regularly and use the resources to help them become more active. Participants left with an information sheet summarizing the discussion. To ensure equal contact time with SW group, participants were sent a generic weekly email reinforcing the physical activity message and to access the MoveMe website for information. This comparison group was chosen over a minimal or no intervention control group because it reflects what could be considered standard practice and provides a test of how the SW intervention compares to this standard practice. The information and support given to the comparison group as well as their receiving a pedometer reflects what is currently available locally for individuals wanting to increase their physical activity.

#### 12-Week Follow-Up

At the end of the 12 weeks, participants met with the researcher and were encouraged to maintain their new level of physical activity over the following 12 weeks, or to continue to try and meet the public health guidelines for physical activity and to continue to use the MoveMe website. Participants kept their pedometer to use during this period if they wished. In this follow-up period, participants were sent 2 emails reinforcing the messages that had been discussed.

### Sample Size

G-Power analysis [49] was used to calculate sample size for between-group analyses of weekly step counts (the primary outcome measure) with repeated measures. Power was set at 0.8, alpha set at 0.05 with a medium effect size (Cohen’s ƒ=.25) expected based on the results of Baker et al [32] who utilized the same pedometer-based goal program. Assuming a correlation among repeated measures of 0.5 with 2 groups and 3 measurements per group, the required total sample size was 86 (43 per group).

### Data Analysis

To ensure the groups were comparable, independent *t*-tests were used to analyze demographic variables and baseline data. The step counts and health variables were analyzed by linear mixed effect models using the R statistical package [50]. Group (2 levels: SW v CP) and time (3 levels: baseline, 12 and 24 weeks) were treated as fixed effects with time a repeated fixed effect. The baseline data and comparison group were used as the reference variable. Participant was a random effect. Several models were conducted (with interaction, without interaction, no random effect, etc) and Akaike Information Criterion comparison was used to assess the best model fit. The main effects and interactions were followed up using general linear hypothesis testing. The blood pressure, cholesterol and/or glucose data from participants who reported taking medications for high blood pressure, cholesterol or Type II diabetes were not used in the analysis of those variables. The motivation data were analyzed using time × group repeated measures ANOVA (MANOVA for barriers to being active) followed up by Bonferroni post hoc tests, the effect size for any effects is denoted by partial eta^2^(*η2*).

## Results

### Participants

**Figure 3:**
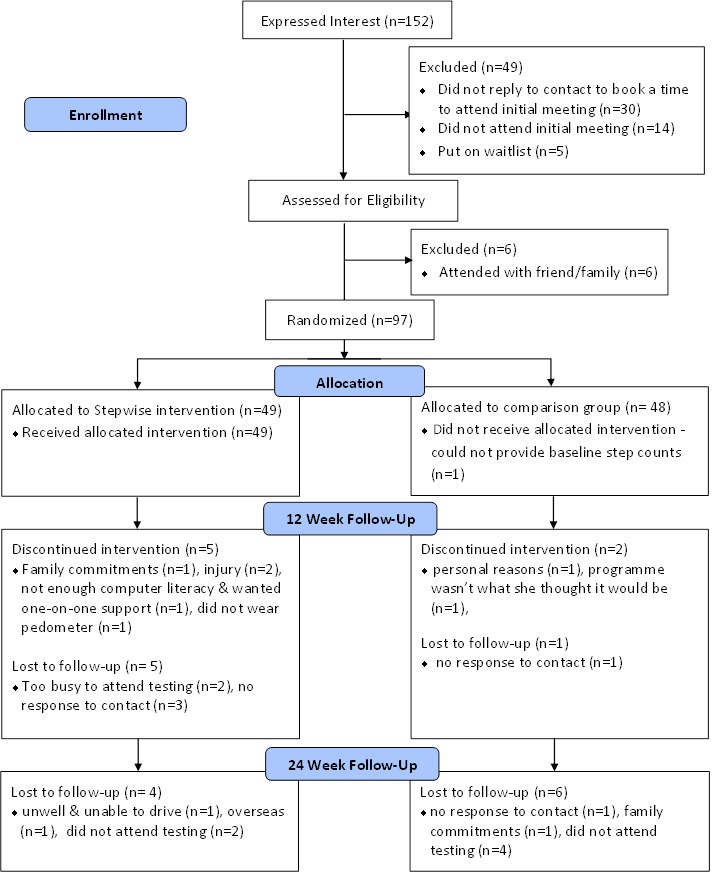
Flow of participants through the study.

Of the 152 people who expressed interest and were eligible to participate, 103 attended baseline testing and 5 people were put on a waiting list (due to funding restrictions, we had a limit of ~100 participants). After baseline testing, 97 participants (82 women, 15 men) were eligible to be randomized, and 42 females and 7 males with a mean age of 47.1 years (SD 11.3) were allocated to the SW group while 40 females and 8 males with a mean age of 45.3 (SD 11.1) were allocated to the CP group (see Figure 3). Participant demographics are shown in Table 1. At baseline testing, 6 participants had to be excluded because they attended with a friend or family member and this compromised the ability to randomly allocate them to a group. Had they been allocated to different groups there would have been contamination between conditions due to the likelihood of them sharing information. Of those who took part in the study, 6 SW and 7 CP participants reported taking medication for hypotension, 1 CP and 1 SW participant were taking medication for high cholesterol and 1 CP participant reported having type 2 diabetes. There were no significant differences between the groups in any of the variables at baseline (see Table 1). Study requirements were completed by 33 SW and 34 CP participants and their data were included in the analysis.

**Table 1:** Participant baseline characteristics^a^.

Baseline			StepWise (n=49) Mean (SD) or n (%)		Comparison (n=48) Mean (SD) or n (%)	
Age (years)			47.1 (11.3)		45.3 (11.1)
Height (cm)			165.8 (7.9)		166.3 (7.8)
Weight (kg)			85.8 (20.2)		85.7 (19.9)
**Ethnicity**					
	NZ European		35 (71)		40 (85)
	Other European		4 (8)		2 (4)
	Maori		2 (4)		2 (4)
	Samoan		2 (4)		1 (2)
	Other		6 (12)		3 (6)
**Employment**					
	Worked full-time		25 (51)		29 (60)
	Worked part-time		10 (21)		10 (21)
	Students		4 (8)		3 (6)
	Homemakers		3 (6)		3 (6)
	Self-employed		3 (6)		1 (2)
	Retired		2 (4)		1 (2)
	Unemployed		2 (4)		—
	Did not report		—		1 (2)
**Education**					
	University degree		30 (61)		36 (75)
	Secondary school		16 (33)		12 (25)
	No secondary school		3 (6)		—
Body mass index			31.2 (6.6)		31.0 (6.5)
Step counts (steps per week)			50,971 (16,069)		53,480 (17,717)
Resting heart rate			66.4 (8.4)		67.5 (10.6)
Diastolic blood pressure (mmHg)^b^			76.6 (9.4)		76.1 (12.9)
Systolic blood pressure (mmHg)^b^			119.5 (15.8)		119.1 (14.9)
Glucose (mmol/L)^c^			5.2 (0.6)		5.4 (0.8)
Total cholesterol (mmol/L)^c^			4.7 (0.9)		4.9 (0.9)
HDL cholesterol (mmol/L)^c^			1.4 (0.4)		1.4 (0.4)
Triglycerides (mmol/L)^c^			1.2 (0.6)		1.3 (0.7)
Positive affect			3.5 (0.7)		3.3 (0.5)
Negative affect			1.5 (0.6)		1.6 (0.5)
Perceived health			71.5 (17.2)		67.3 (14.5)

^a^There were no significant differences between the groups for any of the variables at baseline.

^b^millimeter of mercury

^c^millimoles per litre

### Primary Outcome: Step Counts

**Figure 4:**
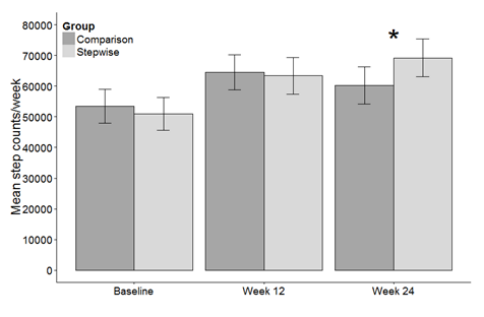
Mean steps per week taken by the SW and CP groups at baseline, week 12 and 24 (* denotes significant difference between groups, *P*=.04).

There were no group differences in step counts between baseline and 12 weeks. All participants increased their step counts per week from baseline taking 11,000 (CI 5739-16,266, *P*<.001) more steps at week 12 and 6,810 (CI 1190-12,431, *P*=.02) more at week 24 (see Table 2 for all significant effects of the intervention). However, an interaction effect showed the change in steps between week 12 and 24 was different between groups, SW took 8939 (CI 274-17,604, *P*=.04) more steps at week 24 than CP (see Figure 4). Separate comparisons for SW and CP showed that SW increased their step counts between week 12 and 24 (CI -11,826 to 112, *P*=.055). CP step counts did not change significantly (*P*=.15).

### Secondary Outcomes

#### Health Related

There were no differences in any of the health-related variables between groups from baseline to week 12 or week 24. However, there were positive physical health changes for all participants across time. There was a mean reduction in triglycerides at week 12 (0.14 mmol/L, CI -0.25 to -0.02, *P*=.02) and decreased diastolic blood pressure (4.22 mmHg, CI -6.73 to -1.72, *P*=.003) at week 12 and week 24 (3.17 mmHg, CI -5.55 to -0.78, *P*=.03). Less positive was an increase in total cholesterol from baseline to week 12 (0.26 mmol/L, CI 0.099-0.423, *P*=.002) and 24 (0.38 mmol/L, CI 0.20-0.56, *P* = <.001) without a concomitant increase in HDL Cholesterol. There were positive effects for psychological health with increased positive affect and decreased negative affect at week 12 and participants perceived they were in better health at week 12 and 24 compared to baseline (see Table 2). There were no significant effects for fasting glucose, weight, resting heart rate or systolic blood pressure.

**Table 2:** Results of the step count and health data for the StepWise (SW) and Comparison (CP) groups at baseline, 12, and 24 weeks.

Outcome Variable		Group	Time			Significant Linear Mixed Model Effects									
			Baseline Mean (SE)	Week 12 Mean (SE)	Week 24 Mean (SE)					β	SE	95% CI			*P*
Weekly Step Counts		SW	50,971 (2747)	63,377 (3031)	69,229 (3176)	Time: Baseline to wk 12				11,002	2685	5739-16,266			<.001
		CP	53,480 (2804)	64,482 (2911)	60,290 (3081)	Time: Baseline to wk 24				6810	2868	1190-12,431			.02
						Time × Group Interaction: wk 12				1403	3895	-6231 to 9036			.72
						Time × Group Interaction: wk 24				11,447	4133	3346-19,548			.006
Total Cholesterol (mmol/L)		SW	4.67 (.12)	4.92 (.13)	4.89 (.14)	Time: Baseline to wk 12				.26	.08	0.10-0.42			.002
		CP	4.91 (.13)	5.17 (.13)	5.29 (.13)	Time: Baseline to wk 24				.38	.09	0.20-0.56			<.001
Triglycerides (mmol/L)		SW	1.21 (.09)	1.11 (.10)	1.10 (.10)	Time: Baseline to wk 12				-.14	.06	-0.25 to -0.02			.02
		CP	1.28 (.09)	1.15 (.10)	1.21 (.10)										
Diastolic blood pressure (mmHg)		SW	76.60 (1.35)	71.68 (2.06)	69.94 (2.62)	Time: Baseline to wk 12				-4.22	1.28	-6.73 to -1.72			.003
		CP	76.13 (1.91)	74.63 (1.90)	73.00 (1.50)	Time: Baseline to wk 24				-3.17	1.22	-5.55 to -0.78			.03
Positive Affect		SW	3.52 (.08)	3.65 (.09)	3.65 (.10)	Time: Baseline to wk 12				.21	.09	0.03-0.38			.02
		CP	3.33 (.08)	3.54 (.09)	3.36 (.09)										
Negative Affect		SW	1.53 (.07)	1.49 (.08)	1.48 (.08)	Time: Baseline to wk 12				-.15	.06	-0.28 to -0.03			.02
		CP	1.55 (.07)	1.39 (.08)	1.42 (.08)										
Perceived Health		SW	71.50 (2.13)	77.62 (2.34)	79.74 (2.43)	Time: Baseline to wk 12				6.37	2.15	2.10-10.65			.004
		CP	67.26 (2.18)	73.63 (2.26)	75.78 (2.36)	Time: Baseline to wk 24				8.52	2.29	3.98-13.06			<.001

####  Motivation

A time main effect showed that task self-efficacy (*F*_1.76,109.13_=9.56, *P*<.001, η^2^= .13) and autonomous motivation (*F*_2,128_=10.12, *P*<.001, η^2^= .14) increased over time while controlled motivation decreased (*F*_1.71,109.71_= 5.80, *P*=.004, η^2^= .08). Compared to baseline, participants felt more confident walking at week 12 (ΔM=9.58, CI 3.09-16.06, *P*=.002) and 24 (ΔM=9.24, CI 2.44-16.05, *P*=.004) had higher autonomous motivation at week 12 (ΔM=0.21, CI 0.06-0.36, *P*=.003,) and week 24 (ΔM=.25, CI 0.10-0.39, *P*<.001,) and had lower controlled motivation at week 24 (ΔM=0.21, CI 0.07-0.35, *P*=.001). There were no differences between the groups.

####  StepWise Website Usage

During the 12-week intervention all but 1 participant self-reported that they had accessed the StepWise website and 84% (28/33) stated they had used it at least once a week, with 15% (5/33) once every 2 weeks. The website usage statistics that were downloaded also confirmed this and showed that 82% of participants (27/33) logged into the website weekly, 1 participant logged in 8 out of 12 weeks, 4 logged in 9 out of the 12 weeks and 1 logged in 10 out of 12 weeks. Encouragingly, all participants used the step log function and therefore automatically received the goal accomplishment and tailored motivation messages and 45% (15/33) regularly used the activity plan function. Together, these results show the participants were engaged with the intervention and used the intervention tools provided on the website. At 24 weeks, self-report data and the website usage statistics showed that 48% of participants (16/33) were still accessing the website with 69% (11/16) accessing it (unprompted by email) at least once every 2 weeks.

#### Features of the SW Website Found to be Most Useful

Participants reported three website features they found most useful: (1) The step goals and pedometer because they provided a weekly challenge, (2) the self-monitoring tools of entering their step count each week and the graph to have a visual representation of the goal and to see whether or not it had been achieved, and (3) the feedback from the motivational messages that provided encouragement and positive reinforcement.

## Discussion

### Principal Results

We developed an intervention containing an individually tailored step goal pedometer walking program, combined with an evidence-based interactive website. In this pilot study we examined whether it would increase walking behavior, improve health and motivation over the short (12-week) and medium-term (24-week) in a community sample of apparently healthy adults. Results showed there were no differences in step counts between the SW and CP groups at week 12, all participants, irrespective of group, increased their step counts from baseline to week 12 and 24. However, importantly, SW participants had significantly higher step counts at week 24 compared to CP participants, suggesting that the intervention successfully helped individuals maintain their new levels of physical activity. In conjunction with the increase in walking, triglyceride levels and diastolic blood pressure improved as well as positive and negative affect (indicators of psychological well-being) and perceived health in participants of both groups. Surprisingly, total cholesterol increased across the study without a significant change in HDL cholesterol. Motivationally, all participants gained greater confidence for walking and their motivation became more autonomous, irrespective of their group assignment. These results are generalizable to a community sample of relatively healthy volunteers wanting to increase their walking behavior.

Participants increased their step counts by 21-24% above baseline at week 12. These results are smaller, but still comparable with, other pedometer interventions with similar samples that have shown increases of around 27% [13-15]. The CP group obtained the same increases in physical activity as the SW group over the short term and a number of components may have contributed to the increase. When participants are motivated enough to enroll in a walking study and know their activity will be monitored this can lead to short term behavior change [14]. Being provided with a pedometer and access to resources about increasing physical activity has also been shown to increase physical activity levels in the short term [51]. If the CP group had not been given a pedometer then, potentially, we would not have seen the same positive increases in physical activity. The strength of the SW intervention is that it resulted in maintenance of behavior change. At week 24 the SW group were achieving, on average, 1277 steps per day more (8939 steps per week) than the CP group and overall averaging 2608 steps per day more (18,256 steps per week) than their baseline values. The CP group achieved 973 steps per day more (6810 steps per week) at week 24 than baseline, and this was a decrease from the positive change of 1571 steps per day (11,002 steps per week) over baseline that they achieved at week 12. Therefore, as expected, the resources provided as the test of standard practice were not enough to maintain behavior change. Only the SW group achieved the goal of the walking program which was to increase their weekly step counts by 15,000 above baseline and meet the physical activity guidelines for health [31]. This is important because few Web-based walking interventions are able to demonstrate (or have not measured) sustained changes in physical activity resulting in the achievement of physical activity recommendations. For example, Carr et al [34] reported their participants’ step counts had returned to near baseline levels at 8 months following their intervention. While a similar study to ours, Carr et al [52] reported that their enhanced Internet group had higher physical activity levels (measured by 7-day physical activity recall) at 12 weeks compared to their standard Internet group (publicly available physical activity websites) but that there were no differences between the groups at 24 weeks. Ideally, future research should continue to monitor behavior change for longer than 24 weeks.

Consequently, despite the lack of group differences at 12 weeks, providing participants with a step goal walking program and access to a website containing the evidence-based behavior change tools to self-monitor their behavior, get feedback and motivational support, and plan their activities, seemed to encourage individuals to maintain the proposed increased physical activity at 24 weeks [6,11-12]. The success of the study was dependent on participants using the website. Encouragingly, both our objective and self-report data showed that 97% engaged the website during the intervention period at least once every 2 weeks. During the follow-up period, when there were no email prompts sent to remind participants to access the website, 48% of participants were still accessing it. These statistics suggest we achieved the goal of creating a website that individuals continued to use. This overcomes one of the reported limitations of Web-based studies, that visits to the website (and therefore exposure and engagement with the intervention) decrease over time [12,37]. Arguably, the content of the website is the key to helping individuals initiate and maintain behavior change. The ability to record steps and see goal achievement was stated as one of the most important features of the website and has been shown previously to be important for successful physical activity behavior change [22-23,52-53]. Alongside the changes in physical activity, there were significant health-related improvements. The decreases in diastolic blood pressure (4.2 mmHg at week 12 and 3.2 mmHg at week 24) were similar to those found with other walking interventions [7,54] and of clinical significance. A 2 mmHg reduction is estimated to reduce the incidence of coronary heart disease by 6% and of stroke by 15% [55]. Our intervention resulted in 0.1% decrease in triglycerides from baseline to week 12. Although this is a modest decrease, a larger effect would not have been expected since our sample had normal levels of triglycerides at baseline [56]. The extent of the decrease in triglycerides required to benefit health remains unclear [57]. The increase in total cholesterol was unexpected, particularly since HDL cholesterol did not change, however, the average values still remain in the normal range [56]. More positively, there were significant improvements in psychological well-being with individuals reporting greater levels of positive affect and reduced negative effect as well as improved perceived health. The change in positive affect and perceived health was also shown by Fitzsimons et al [33] who employed the same step-goal-based walking program but in a non-Web-based setting.

The study was successful in changing motivation for physical activity in both groups. Participants felt more confident in their ability to walk for longer periods of time. Increased confidence has been demonstrated in a number of walking studies and is a strong predictor of physical activity behavior [58]. The personal performance successes participants could see from their step counts increasing (both groups) and achieving goals (SW group) would have contributed to the increased confidence [58-59]. Participants had greater autonomous motivation at the conclusion of the study compared to the baseline, meaning that they were more motivated to participate in physical activity out of enjoyment and for the value they attached to the outcomes of being active. Furthermore, they had less controlled motivation at the end of the study meaning they were not feeling as pressured to participate in activity. Together these changes mean individuals are more likely to *want* to be physically active rather than feeling they *have* to be physically active and the changes are related to increased and sustained physical activity behavior [60]. Interestingly, the change in controlled motivation did not occur until week 24 when participants had completed the intervention. It may be that the act of being involved in a pedometer-based study which required self-monitoring of behavior (SW) or prompted individuals to decide to self-monitor (CP) can act as a controlling pressure on behavior. The impact of pedometers on autonomous and controlled motivation has not been investigated and would be an interesting avenue for future research, to ensure pedometer-based interventions do not have a negative effect on motivational quality.

### Limitations

Both groups received a pedometer and physical activity device, therefore the study did not have a no treatment control group. Consequently, there is still the possibility that some other factor other than the elements contained in the intervention and comparison group caused the change in physical activity behavior. Step counts were only recorded for analysis at baseline, 12 weeks and 24 weeks. It may be that participants simply increased their walking behavior in the measurement week and these values do not actually reflect the activity they achieved during the intervention. However, for someone to have such a large increase in activity in one week is unlikely. The walking program automatically increased weekly step count goals, so consequently if something happened to disrupt (decrease) normal walking patterns (eg, getting ill) then the individual would be expected to meet a new, harder goal on resumption of the program. Ideally, the person would have been prompted by the program to go back to the walking goal from the week prior to the disruption. However, our StepWise software was not able to do this. This should be taken into account in future development of the intervention. Additionally, the intensity at which participants walked was not measured. Although we endeavored to emphasize that participants walked at moderate intensity, we cannot be sure they adhered to this. It was hoped that the study would have 80% power to detect differences in step counts (the primary outcome), however, after drop-outs we did not achieve our target sample size of 43 per group. Our large drop-out rate is a limitation; we were able to elicit reasons for why 9 SW and 3 CP participants decided not to continue with the study but we were not able to ascertain the reasons why 5 SW and 6 CP did not show to the testing sessions. As a result, we cannot be sure whether there was some underlying factor that caused these individuals to drop out and therefore bias the results. Furthermore, it is likely the study was underpowered to detect differences in the biochemical health measures taken. Finally, in hindsight, it would have proved useful to also ask participants if there were any other features they would have liked the website to have to encourage and support their physical activity. This would have been advantageous for future development of the StepWise website.

### Conclusions

There were no differences in step counts between the SW and CP groups following the 12-week intervention; all participants increased their walking behavior and had improved physical and psychological health. However, only the StepWise group successfully maintained their increased walking behavior 3 months post-intervention and achieved step counts that met the physical activity guidelines for health. Sustained behavior change is supported by a step-goal-based walking program with behavior change tools that allow a person to self-monitor behavior, get feedback and motivational support, and make activity and coping plans.

## Appendix

Multimedia Appendix 1 CONSORT EHEALTH form V1.6.

## Abbreviations

BCT: behavior change technique
CP: comparison group
HDL: high density lipoprotein
SW: StepWise intervention group
